# Synaptic and Fast Switching Memristance in Porous Silicon-Based Structures

**DOI:** 10.3390/nano9060825

**Published:** 2019-05-31

**Authors:** Vicente Torres-Costa, Ermei Mäkilä, Sari Granroth, Edwin Kukk, Jarno Salonen

**Affiliations:** 1 Deptartamento de Física Aplicada and Centro de Micro-Análisis de Materiales, Universidad Autónoma de Madrid, Cantoblanco, 28049 Madrid, Spain; 2 Department of Physics and Astronomy, University of Turku, FI-20014 Turku, Finland; ermei.makila@utu.fi (E.M.); sari.granroth@utu.fi (S.G.); edwin.kukk@utu.fi (E.K.); jarno.salonen@utu.fi (J.S.)

**Keywords:** porous silicon, memristors, resistive switching, synaptic emulation

## Abstract

Memristors are two terminal electronic components whose conductance depends on the amount of charge that has flown across them over time. This dependence can be gradual, such as in synaptic memristors, or abrupt, as in resistive switching memristors. Either of these memory effects are very promising for the development of a whole new generation of electronic devices. For the successful implementation of practical memristors, however, the development of low cost industry compatible memristive materials is required. Here the memristive properties of differently processed porous silicon structures are presented, which are suitable for different applications. Electrical characterization and SPICE simulations show that laser-carbonized porous silicon shows a strong synaptic memristive behavior influenced by defect diffusion, while wet-oxidized porous silicon has strong resistance switching properties, with switching ratios over 8000. Results show that practical memristors of either type can be achieved with porous silicon whose memristive properties can be adjusted by the proper material processing. Thus, porous silicon may play an important role for the successful realization of practical memristorics with cost-effective materials and processes.

## 1. Introduction

Memristors were first described in 1971 by Leon Chua as the fourth basic circuit element [1]. They are two terminal electrical devices whose conductance depends on the total amount of charge that has crossed it. As a consequence, these devices have memory of their bias history, hence the term memristor, i.e., memory resistor. Since then, many applications have been foreseen for memristors, such as two-terminal resistive memory cells (ReRAM) [2] or logic function implementation [3]. Resistive memories based on memristors have attracted a lot of attention in recent years due to the possibility of implementing large memory capacity with simple two terminal memory cells, which would allow a simplification of the circuitry layout and at the same time increase the integration scale of memory chips [4]. Another exciting application of memristors is the development of neuromorphic systems [5], due to some similarities of memristors with neural synapses. Since Strukov et al. first reported a TiO_2_ memristor in 2008 [6], most structures reported to show memristive properties are based on metal oxide nanowires or nanolayers [7], which makes their implementation in current technology challenging. The development of low cost, industry-compatible memristive materials is of capital importance for the realization of functional memristonic systems. Here we present the realization of all-silicon memristors with synaptic and resistance switching properties with potential applications in synapse emulation and neuromorphic systems and implementation of silicon ReRAM memory cells, respectively.

## 2. Materials and Methods

Thermally hydrocarbonized porous silicon (HCPSi) structures were fabricated by the electrochemical etch of 3” monocrystalline Si wafers in an hydrofluoric acid-based electrolyte and subsequently hydrocarbonized by acetylene decomposition following a well-established procedure (see for example Ref. [8]). The anodization current varied from 50 mA/cm^2^ to 100 mA/cm^2^ with etching times of 30–100 s, which results in a spongelike morphology with a porosity between 55 and 65%, pore sizes around 50 nm, and photoluminescent nanocrystals around 5 nm [9,10]. To produce laser-carbonized (LaCPSi) structures, samples were cut into 10 × 10 mm^2^ pieces and placed in a transparent reactor cell at ambient temperature under an acetylene flow of 15 sccm. Carbonization provides effective passivation of porous silicon, while retaining its porosity [11]. Local carbonization of the surface was achieved by laser-induced acetylene decomposition using a focused 5 W beam (450 nm wavelength) and a computer-controlled X-Y stage, resulting in 3 × 3 mm^2^ laser-carbonized porous silicon pads surrounded by HCPSi, on silicon. To produce wet-oxidized carbonized (wetOxCPSi) PSi samples, 15 × 15 mm^2^ HCPSi samples were introduced into a tube furnace and treated at 800 °C for 3–6 h under humid N_2_ flush (1.5 L/min). Both kind of devices were completed by providing top and bottom electrodes by either an e-beam evaporated indium-tin oxide (ITO) layer with subsequent thermal annealing or sputtered Au electrodes deposited through an 1.5 × 1.5 mm^2^ mask, or simply with conductive silver paste. The type of contact did not show any influence on the measurements.

Electrical measurements were carried out using a Bio-Logic Science Instruments (Seyssinet-Pariset, France) SP-150 potentiostat/galvanostat, considering the bottom contact as the positive electrode. 

## 3. Synaptic Memristance

Local laser irradiation of hydro-carbonized porous silicon in an acetylene atmosphere leads to the formation of a carbonized porous silicon pad on the silicon substrate. A clear local change in hydrophobicity (HCPSi is hydrophobic whereas CPSi is hydrophilic) is observed by the naked eye after irradiation, confirming the change in surface chemistry. A detailed X-ray photoelectron microscopy (XPS) characterization is available in the Supplementary Materials S1.

### 3.1. Electrical Properties

**Figure 1:**
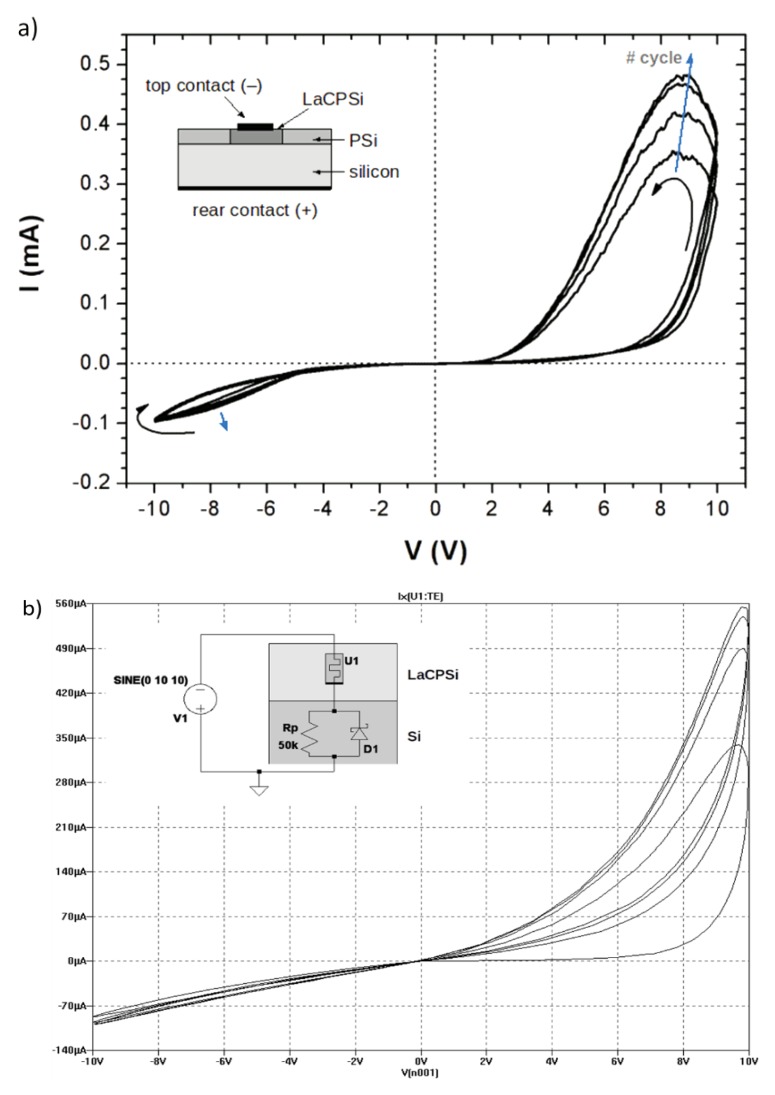
(**a**) I-V plot of a laser carbonized porous silicon (LaCPSi) structure. Voltage was swept sinusoidally with a 10 V amplitude at a rate of 5 V/s. Arrows indicate the voltage path. The pinched hysteresis loop typical of memristors is evident. The inset shows an schematic view of the device. (**b**) LTSpice simulation of a LaCPSi/Si structure. The inset shows the equivalent circuit used for the simulation.

The laser-carbonized porous silicon/silicon (LaCPSi/Si) structure exhibits a hysteresis current-voltage loop indicative of memristance (Figure 1a). The device shows a rectifying behavior, with higher conductance at forward bias, as in conventional porous silicon/silicon junctions [12]. However, in this case, the conductance at any given bias depends on the followed bias path, or polarization history, which demonstrates the memory effect of the LaCPSi memristor. In contrast with most memristors based on metal oxides, a first electroforming step was not necessary in this case. Also, the resistance changes from the high resistance state to the low resistance state in a gradual fashion rather than abruptly, without a characteristic switching voltage [2,13]. Still, the device’s resistance spans more than one order of magnitude. Figure 1a shows the I-V performance of one of the memristors when swept in quasi-static conditions (125 mHz) with an amplitude of 10 V. From the plot, it can be seen that at 5 V bias, resistance changes from ca. 590 KOhm to 30 KOhm depending on whether the bias voltage is increasing or decreasing, yielding a switching ratio of 20. This result suggests that LaCPSi are flux-controlled memristors, since their I-V characteristics follow the modified Ohm’s law:

I(t) = G(φ(t))·V(t)(1)

where G(φ(t)) is the memconductace (the inverse of memristance), and the flux-linkage φ(t) is the integral of V(t) over time [1].

To better understand the memristive properties of LaCPSi, the device was simulated in LTSpice IV. The silicon/LaCPSi interface was modeled as a Schottky diode with a parallel resistor to account for poor rectification, and the LaCPSi layer was simulated using the memristor model developed by Chang et al. [14]. This model considers that conduction arises from a parallel combination of Schottky barrier emission and tunneling at the memristor/electrode interface, with field-driven ion drift modulating their relative contribution. Figure 1b shows the simulations results after optimizing the model’s parameters. It can be observed that there is reasonable good agreement with the experimental measurements, which suggests that a field-modulated parallel combination of Schottky and tunneling conduction, which results in areal-type memristance, may be responsible for the memristive properties of LaCPSi, as assumed in Chang’s model [14]. It must be mentioned that in order to achieve a good agreement with experimental data, the optional diffusion term in Chang’s model had to be included in order to achieve the overlapping of successive loops observed. This term accounts for high mobility vacancies which, in addition to field-driven drift, are also able to diffuse back to their ground high resistance state, which explains the overlap of successive I-V loops [14]. This indicates that high mobility defect diffusion plays an important role in LaCPSi memristance [15,16].

### 3.2. Synaptic Memristance in LaCPSi

The conductance of the LaCPSi structure depends, in the end, on the total charge that crossed the device. This behavior is similar to that of a neural synapse. In a biological system, the synaptic strength of a synapse (i.e., the ability or efficiency to transfer neurotransmitters from one neuron’s axon to another neuron’s dendrite) depends mainly on the total flux of Ca^2+^ ions released across the synaptic membrane by electrochemical pulses (called “synaptic spikes”). [17] Depending on the polarity, intensity and number of spikes, the synaptic strength is enhanced or reduced, in a process called potentiation or depression, respectively. In this way, the repeated application of potentiation spikes reinforces the connection between neurons, while repeated depression pulses deteriorates it. This ability to modulate the synaptic strength by the repetitive application of the proper stimulus is called synaptic plasticity and constitutes the basis of biological learning, long term memory and “brain programming” [18]. The possibility to artificially imitate this behavior would lead to neuromorphic computing systems that, by means of emulating the working principles of nervous tissue, would have important advantages over traditional digital systems in complex non-numeric tasks, distributed processing, adaptive learning, fault tolerance, fuzzy logic, and reconfigurable design, among others [19,20].

**Figure 2:**
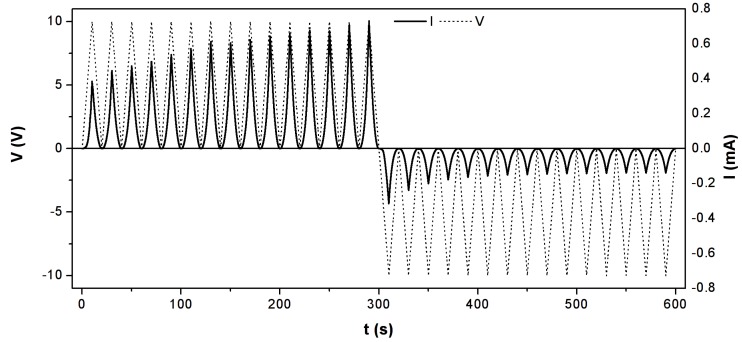
LaCPSi potentiation/depression pulses. Current across a LaCPSi memristor measured during successive positive (negative) bias ramps. The potentiation (depression) effect on the device’s conductance of the positive (negative) pulses can be observed as the sequential increase (decrease) of current after every ramp.

In order to investigate the synapse emulation capabilities of the LaCPSi memristors, the devices were subjected to successive voltage ramps (i.e., excitation spikes) and the current intensity was recorded. Results are shown in Figure 2. It can be clearly seen how conductance gradually increases (is potentiated) after every positive spike and how it decreases (is depressed) with every negative spike, showing a synaptic-like plasticity.

**Figure 3:**
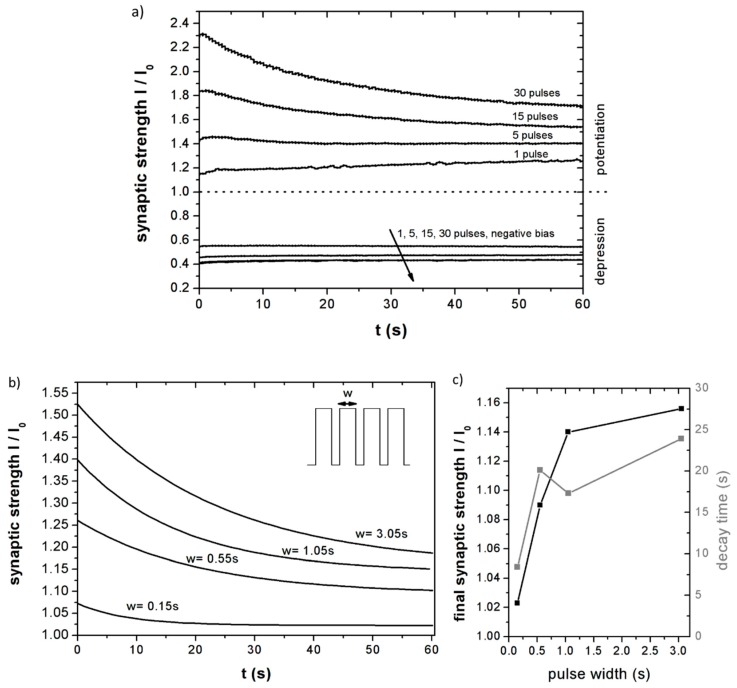
(**a**) Relative current across a LaCPSi memristor after stimulation with an increasing number of potentiation pulses. Current at a 2 V bias of a LaCPSi memristor once the stimuli has ceased. The current value is normalized to the value before stimulation, thus representing the ‘synaptic strength’ of the ‘synapse’. Stimulus consisted of different number of 10 V pulses of either polarity. (**b**) Dependence of synaptic strength as a function of pulse duration. Synaptic strength of a LaCPSi memristors at 0.5 V bias after stimulation with 10 potentiation pulses of 10 V of increasing width. Pulse separation was 50 ms. (**c**) Dependency of the final synaptic strength and decay time of LaCPSi memristors with potentiation pulse width.

To further study the synaptic plasticity of the porous silicon memristors, a series of excitation pulse trains were applied to the devices, and afterwards the current was measured and normalized to the value measured before excitation in order to determine the relative change in conductance, that is, its synaptic strength. The actual measurement protocol, as well as a complete sequence of stimulation and readout is available in the Supplementary Materials S2. Figure 3a summarizes the results obtained after stimulation with an increasing number of 10 V pulses of either polarity at a readout voltage of 2 V.

Results clearly show that positive pulses lead to a potentiation of the synaptic strength (conductance), while negative ones provoke its depression. It is also worth noting that the effect of potentiation/depression increases with the number of pulses, that is, with the stimulation amount, although this effect is less prominent in the depression process. In the case of the strongest potentiation, conductance was enhanced up to 230%, while the most intense depression reduced the conductivity of the memristor down to 45% of its initial value. Figure 3a also shows that for more insistent stimulation (15 and 30 pulses), conductance decays in time after stimulation, while in the case of 1 and 5 pulses it increases slightly. This effect is attributed to the relatively high read voltage used in this particular experiment: The 2 V read voltage is comparable to the 10 V excitation pulses, which may cause by itself a potentiation effect during readout, thus not only cancelling the synaptic strength decay, but rather making it increase.

To cancel out this effect and better study the decay of the synaptic strength of the LaCPSi memristors, a different set of measurements was performed. In this case, the stimuli consisted of 10 V pulses 50 ms apart, of increasing duration. The current was measured at 0.5 V in order to minimize the readout effect. As it can be derived from Figure 3b, all stimulation patterns potentiated the synaptic strength, with higher potentiation achieved when applying wider excitation pulses. In the case of 3 s pulses, the device’s conductivity increased by more than 50%.

Once the excitation ceases, the synaptic strength decays in all cases. A first order exponential fit allows the determination of both the decay time and the short-term (initial) and long-term (final) synaptic strength. Both parameters are summarized in Figure 3c. Decay time for this stimulation pattern ranges from 8.4 s for 150 ms pulses to 23.9 s for the longest (3 s) pulses, which indicates that more intense stimulation has a longer lasting effect on the memristor. This effect is also present in biological synapses, is related to short/long term memory and learning/forgetting mechanisms [18]. and is crucial to achieve a neuromorphic system that successfully mimics the synaptic adaptive functions of neural systems.

**Figure 4:**
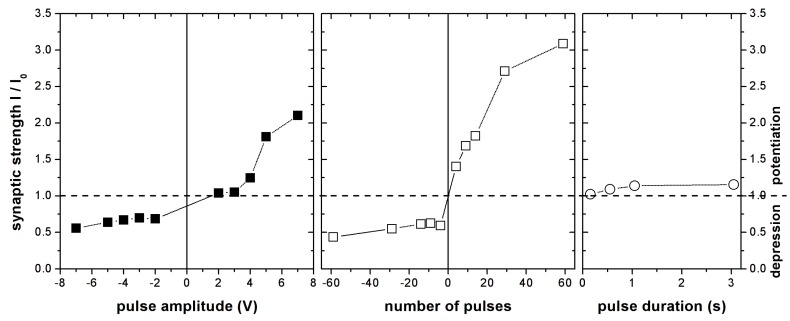
Synaptic plasticity of LaCPSi memristors. Summary of the potentiation/depression effect of different stimulation patterns on LaCPSi synaptic memristors. Different amounts of potentiation and depression can be achieved using the appropriate excitation pattern.

Synaptic strength may also be adjusted by controlling the stimuli amplitude. To verify this, LaCPSi memristors were stimulated with a train of nine pulses (100 ms wide, 50 ms apart) of different amplitudes of either polarity. Again, positive bias pulses induced a potentiation of the synaptic strength, while negative bias depressed it. With this stimulation pattern, maximum potentiation reached 2.1 with 7 V pulses, while the highest depression reached 0.56 at −7 V. Figure 4 summarizes the relative change in conductance of the LaCPSi memristors for this and other stimulation patterns, showing that the conductance (synaptic strength) can be gradually adjusted within a wide range of values, between 0.4 and 3 of its initial value, by exciting the memristors with the adequate stimuli.

## 4. Resistive Switching Memristance

Wet-oxidized carbonized porous silicon (wetOxCPSi) is interesting material mainly because of its broad, tunable, white photoluminescence (PL) which has very different properties compared to other oxidized PSi structures [21]. Although the origin of this PL is not clear, there are some evidences that it results from carbon clusters formed during the treatment [22,23]. XPS characterization of wetOxCPSi samples is provided in Supplementary Materials S1.

Silicon oxide has been proven to show rapid resistive switching due to the formation and destruction of conduction pathways composed of silicon inclusions across the oxide film [24]. Here, HCPSi was subjected to wet oxidation in order to achieve a complete oxidation of the porous matrix, hence optimizing the switching properties of the structure.

### 4.1. Electrical Properties

**Figure 5:**
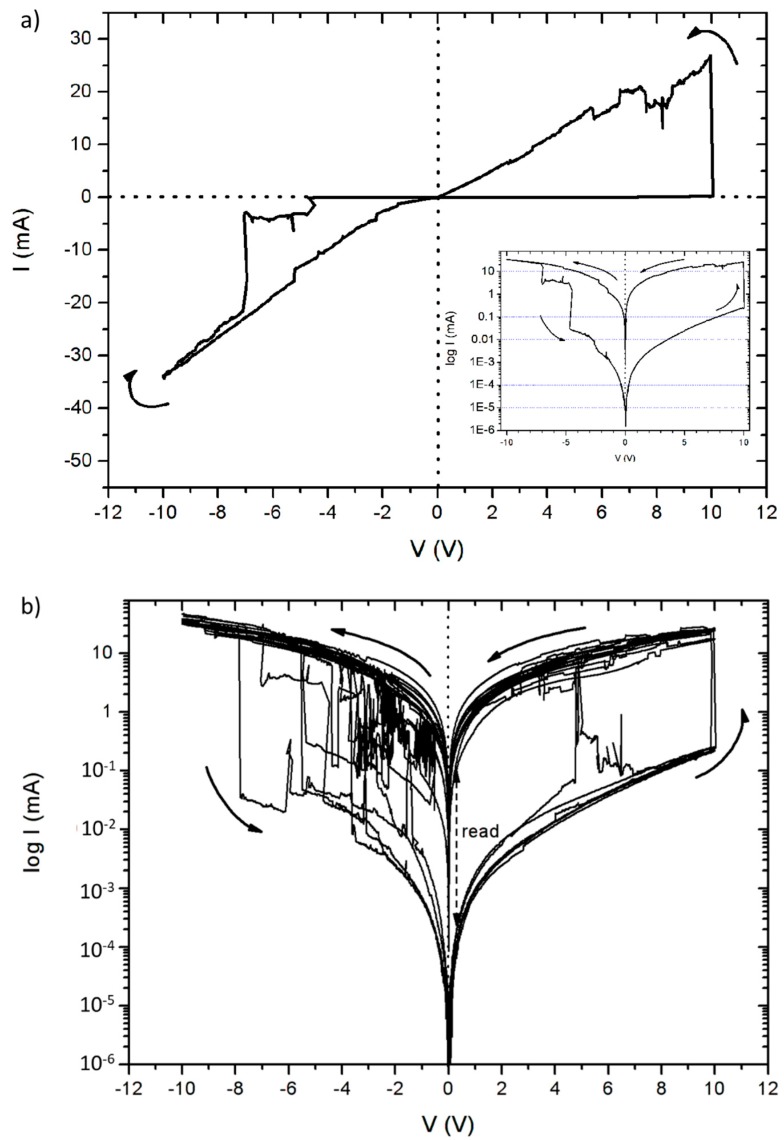
(**a**) I-V plot of a wet-oxidized porous silicon (wetOxPSi) structure. Voltage was swept from 0 V to 10 V, to −10 V and back to 0 V at a rate of 5 V/s. Arrows indicate the voltage path. The memristive hysteresis loop and abrupt resistance switching are evident. Inset: log plot of the same data. (**b**) Eleven successive I-V loops in the +10 to −10 V range of a wetOxPSi memristor.

After wet oxidation, the electrical properties of the porous silicon structures changes drastically. Figure 5a shows a characteristic I-V curve of a wetOxCPSi device completed with a top ITO contact Voltage which varied at a sweep rate of 5 V/s. No compliance current was set to limit the current consumption of the device, in order to obtain the full I-V characteristics of the device, although this implies a high power consumption (for an electronic component). As it can be observed, the wetOxCPSi behaves as a bipolar memristor, with strong resistance switching at both positive and negative biases. At low positive bias, the device is in the high resistance state (HRS) until a certain threshold voltage is reached. At that moment, the memristor switches to a low resistance state (LRS) allowing currents of several milliamps. Once in the LRS state, the device remains in this state at either polarization, until a reverse bias threshold is reached, after which the memristor switches back to the HRS state. The transition between the states is less than 30 ms, which is the sampling rate of the experimental setup. The log-plot of the curve shown in the inset allows to examine the part of the I-V curve corresponding to the high resistance state, and at the same time, reveals that the hysteretic loop is pinched at 0 V, proving the memristive behavior. At a forward bias of 3.5 V, the high resistance state and low resistance state are 356 KΩ and 354 Ω, respectively, resulting in a switching ratio (SR) of around 1000. The switching ratio increases at lower bias (<0.5 V) in either polarization, reaching a maximum value of 8064 at 0.2 V forward bias. The diffusion of oxygen vacancies driven by the applied electric field is believed to be the fundamental mechanism of strong filamentary resistive switching in silicon oxide films [24,25,26,27]. The diffusion of oxygen vacancies favors the phase separation of the film, inducing the nucleation of silicon nanoclusters at oxygen vacancies [24,27]. This phase separation is further favored by structural defects present in the oxidized porous silicon matrix.

WetOxCPSi memristors were subjected to sequential I-V cycles from −10 to 10 V in order to check the resistive switching over time. Figure 5b shows the results of one of these experiments. No electroforming step was necessary before the experiment. First, it can be observed that the resistive switching is achieved in every cycle, although at reverse bias, the switching is rather erratic and noisy. The switching “reset” voltage in reverse bias (the voltage that triggers the LRS to HRS transition) may be anywhere between −8 and −1 V, although the switching ratio is still of three orders of magnitude in any case. At forward bias, however, the behavior is much more stable, with a well-defined “set” voltage (HRS to LRS transition) of around 9.8 V. It can be observed that a clear gap between the HRS and LRS states below +4 V is clearly visible in every cycle.

### 4.2. Switching Memristance in WetOxCPSi

**Figure 6:**
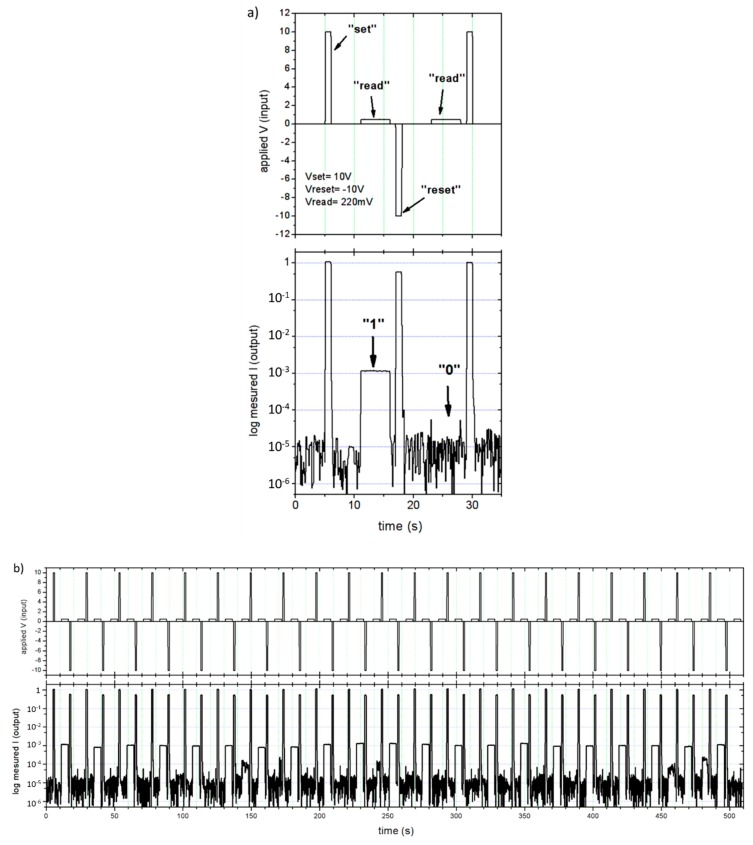
(**a**) Description of the read/write operation of a wetOxPSi memristor. (**b**) Successive read/write operations on a wetOxPSi ReRAM cell.

Read/write experiments were performed on wetOxCPSi memristors in order to study their suitability as ReRAM devices. Figure 6a shows the measurement procedure: First, a 10 V set pulse is applied for 1 s to put the memristor in the low resistance state (i.e., store an “1” bit in the ReRAM cell). Then, the resistance is read by measuring the current at 220 mV forward bias. In the next cycle, a reset pulse of −10 V for 1 s is applied to store a “0” bit, and the resistance is read again at the same read voltage of 220 mV. Between each step, the memristor is shorted (0 V bias) in order to remove any possible charge stored in parasitic capacitances at the device’s contact. The set and reset pulse duration was chosen for practical reasons for acquisition. Proper switching was also achieved with pulses of less than 0.5 s. Figure 6b shows a sequence of read/write processes. It can be observed how the bit written to the memristor is clearly defined in each write process, with a difference of two orders of magnitude in resistance between the 0 (22 KΩ) and 1 (220 Ω) binary levels. No apparent degradation of the performance is observed for more than 1 h. The last stored value is clearly readable after more than 24 h of leaving the device unattended, indicating a very persistent and stable switching (not shown). Due to the low read voltage used, the current measured after a reset operation, corresponding to the “0” level, is barely above the noise floor level of the device. This would facilitate the design of the read circuitry of a wetOxCPSi ReRAM cell, since it is not necessary to distinguish between two current levels: Just the detection of some current above the noise level is indicative of an “1” stored in the cell.

## 5. Conclusions

Porous silicon is a promising material for the future development of novel silicon-based memristive structures which may show a broad range of applications. Results have shown that with the proper modification, e.g., laser-induced carbonization and wet oxidation porous silicon can exhibit different memristive properties suitable for different kinds of memristive applications.

SPICE simulations show that laser carbonized porous silicon structures behave as flux-controlled memristors, whose conductance depends on the total charge that crossed the device, that is, on the past polarization history of the structure. Conductance is modulated by field-driven defect drift, but also by defect diffusion inside the structure, resulting in areal-controlled memristance. In addition, results indicate that these memristors show synaptic properties such as plasticity and short/long term memory. The conductance of these memristors can be gradually adjusted, increased or decreased, within a wide range of values by applying the proper stimulation, which, much akin to the synaptic strength between two neurons is modulated by excitation spikes across the synapse. Thus, the carbonized porous silicon structures presented in this work can serve as very promising, CMOS industry-compatible, synaptic memristors for the development of electronic neuromorphic computing systems that successfully emulate biological neural structures.

On the other hand, wet oxidized porous silicon exhibits strong filamentary-type resistance switching. Switching ratios above 8000 have been achieved in this work. These porous silicon memristors have been used as two terminal resistive memory cells, in which the ability to store data bits in the form of material resistance and its latter retrieval in simple read/write processes has been demonstrated. The possibility to operate these memory cells in a two terminal configuration and the fact that one of the logic levels stored in the cell lies below the noise level, opens the way to use these wet oxidized porous silicon memristors in ReRAM chips of increased integration level and simplified circuitry layout.

In all, this work shows the possibilities offered by porous silicon to achieve different memristive characteristics. By properly tailoring these characteristics, a wide variety of devices can be developed and implemented in a new generation of electronic and memristonic systems.

## Supplementary Materials

The following are available online at https://www.mdpi.com/2079-4991/9/6/825/s1, Figure S1.1: XPS spectra of Si 2p and C 1s peaks from a LaCPSi pad and wetOxCPSi. Figure S1.2: XPS spectra of Si 2p (left) and C 1s (right) peaks from a wetOxCPSi. Figure S1.3: Water drops on a hydrophobic HCPSi substrate and on a hydrophilic LaCPSi pad. Optical microscopy image of an intact LaCPSi pad after the as-anodized porous silicon has been dissolved away with 1 M NaOH. Figure S2: Procedure of the stimulation experiments on LaCPSi. Table S1: XPS results of a laser carbonized pad and the thermally hydrocarbonized PSi background.
