# Supplementary material for: Synaptic and Fast Switching Memristance in Porous Silicon-Based Structures
Source: Nanomaterials (Basel). 2019 May 31;9(6):825. doi: 10.3390/nano9060825 (PMC6631600; doi:10.3390/nano9060825)

Synaptic and fast switching memristance in porous silicon-based structures

V. Torres-Costa[1], E. Mäkilä[2], S. Granroth[2], E. Kukk[2] , J. Salonen[2]

Supplemental Material

S.1 XPS characterization of LaCPSi and wetOxCPSi

Chemical characterization was carried out by X-ray Photoelectron Spectroscopy (XPS). Measurements were performed with a PHI 5400 ESCA spectrometer (PerkinElmer Co., USA) with a Mg K_α x-ray source ($h\upsilon$=1253.6 eV). Survey spectra were collected with pass energy of 89.45 eV and the core level spectra with a pass energy 35.75 eV. Peak fitting was done using Gaussian-Lorentzian line shapes with a Shirley type background.

XPS was used to study the chemical changes on HCPSi after laser irradiation in acetylene atmosphere. Figure S1.1 shows the experimental results and deconvolution fits of a LaCPSi pad, while Table S1 summarizes the results of HCPSi before and after the laser carbonization process. HCPSi shows different kinds of hydrocarbon species, and the low ratio of Si-C/C=C bonds and the larger amount of C-C and Si-Si bonds suggest that carbon is not diffused deep into the silicon porous structure. After laser carbonization however, the amount of Si-C and =-Si-C bonds increase, while C-C and Si-Si bonds decrease, indicating the incorporation of carbon to the silicon structure. Due to the hydrogen desorption during laser carbonization and the formation of the obligatory oxide termination on the LaCPSi surface, the amount of SiOx and C-O-H bonds also increase after the laser process. These results are in agreement with what is expected when the loose hydrocarbon layer on top of HCPSi starts to form a non-stoichiometric oxide-terminated SiC layer in a successful laser carbonization process.

These changes in the surface chemistry lead also to a clear change in the hygroscopicity of LaCPSi compared to that of HCPSi. Figure S1.3 shows the evident difference in water contact angle on HCPSi and LaCPSi. Results also show an efficient local laser carbonization on as-anodized, hydrogen terminated porous silicon. As for further evidence of a successful carbonization, the sample was placed in a 1M NaOH solution which rapidly dissolved the as-anodized part of the sample, but left the LaCPSi pad intact (Figure S1.3-right).

The wet oxidation process of HCPSi is very dependent on the treatment temperature and atmosphere, and need to be done in total absence of dry O_2 (usually in N_2 or in Ar). In dry O_2 treated CPSi is

completely black lacking all the photoluminescence properties. Although the origin of the PL is not clear, there are some evidences that it results from carbon clusters formed during the treatment [17,18]. Our XPS results (Figure S1.2) also support this showing almost completely oxidized Si structure (Si (2p) and O(1s) peaks), but still clearly detected amount of C-C bonds associated with C (1s) peak. Although some of the detected carbon may be due to adventitious carbon, the wetOxCPSi samples showed characteristic PL that cannot originate from a pure SiO_2 structure.

Table S1.

XPS results of a laser carbonized pad and the thermally hydrocarbonized PSi background.

	Si-C/C=C (%)	C-C (%)	C-O-H (%)	C=O (%)	Si-Si (%)	Si-C (%)	=-Si-C (%)	SiO_x (%)
HCPSi background	**18**	**63**	**14**	**6**	**58**	**11**	**9**	**22**
LaCPSi pad	**20**	**58**	**16**	**6**	**48**	**18**	**10**	**26**

Figure S1.1

XPS spectra of Si 2p (left) and C 1s (right) peaks from a LaCPSi pad.

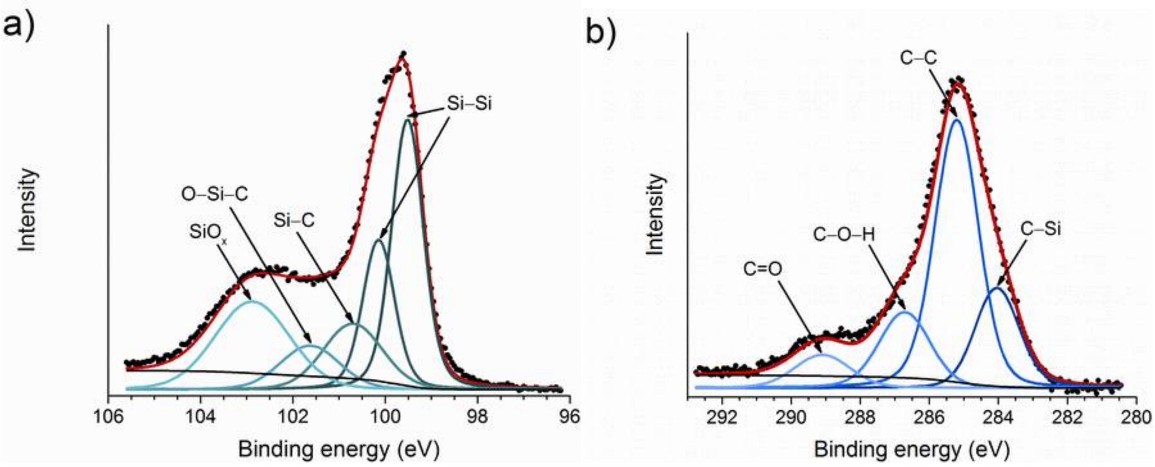

Figure S1.2.

XPS spectra of Si 2p (left) and C 1s (right) peaks from a wetOxCPSi.

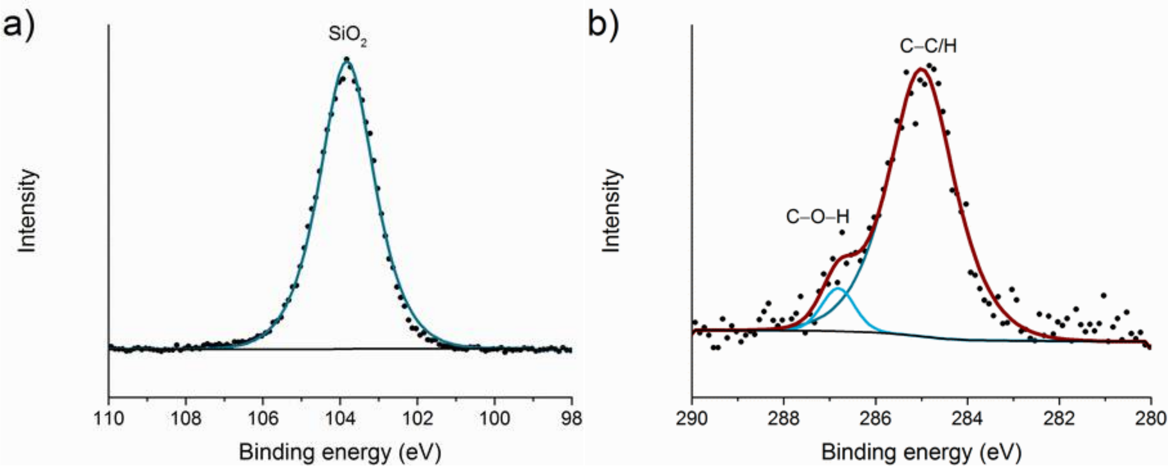

Figure S1.3.

Water drops on a hydrophobic HCPSi substrate (left) and on a hydrophilic LaCPSi pad (center). Optical microscopy image of an intact LaCPSi pad after the as-anodized porous silicon has been dissolved away with 1 M NaOH. (right)

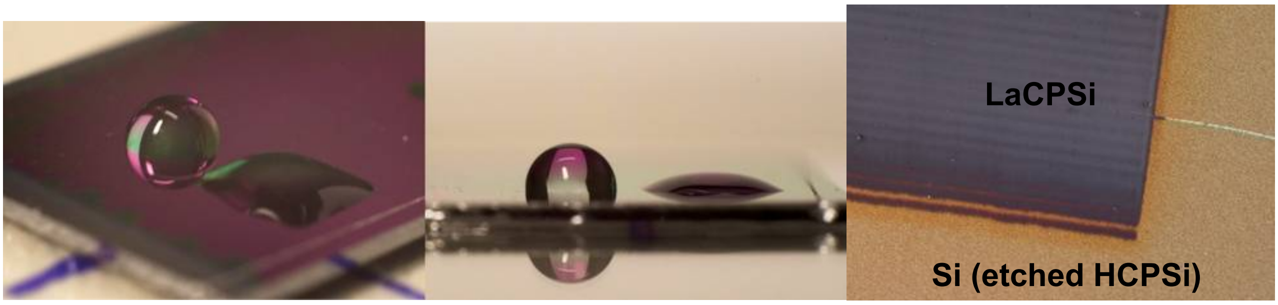

S2. Stimulation experiments on LaCPSi memristors.

The stimulation and readout procedure is depicted in Figure S2. Left: Before stimulation, samples are "initialized" by applying a strong reverse bias pulse in order to remove any possible effect of previous stimulation (*reset*). Then the reference conductance is determined by measuring the current at a given read bias (*read I_0*), Stimulation pulses are then applied, of positive bias for potentiation, or reverse bias for depression (*stimulus*). After stimulation, current is measured again at the same read bias as the reference for a given period of time (*read I*). The relative conductance change I/I_0 gives the amount of potentiation/depresion achieved. Figure S2-Right: Complete stimulation/readout sequence of a stimulation experiment consisting of increasing number of potentiation pulses. The negative current values during stimulation shown in the plot are measurement artifacts due to the sudden application and removal of the 10V pulses. Current was actually not recorded during reset and stimulation.

Figure S2: Procedure of the stimulation experiments on LaCPSi.

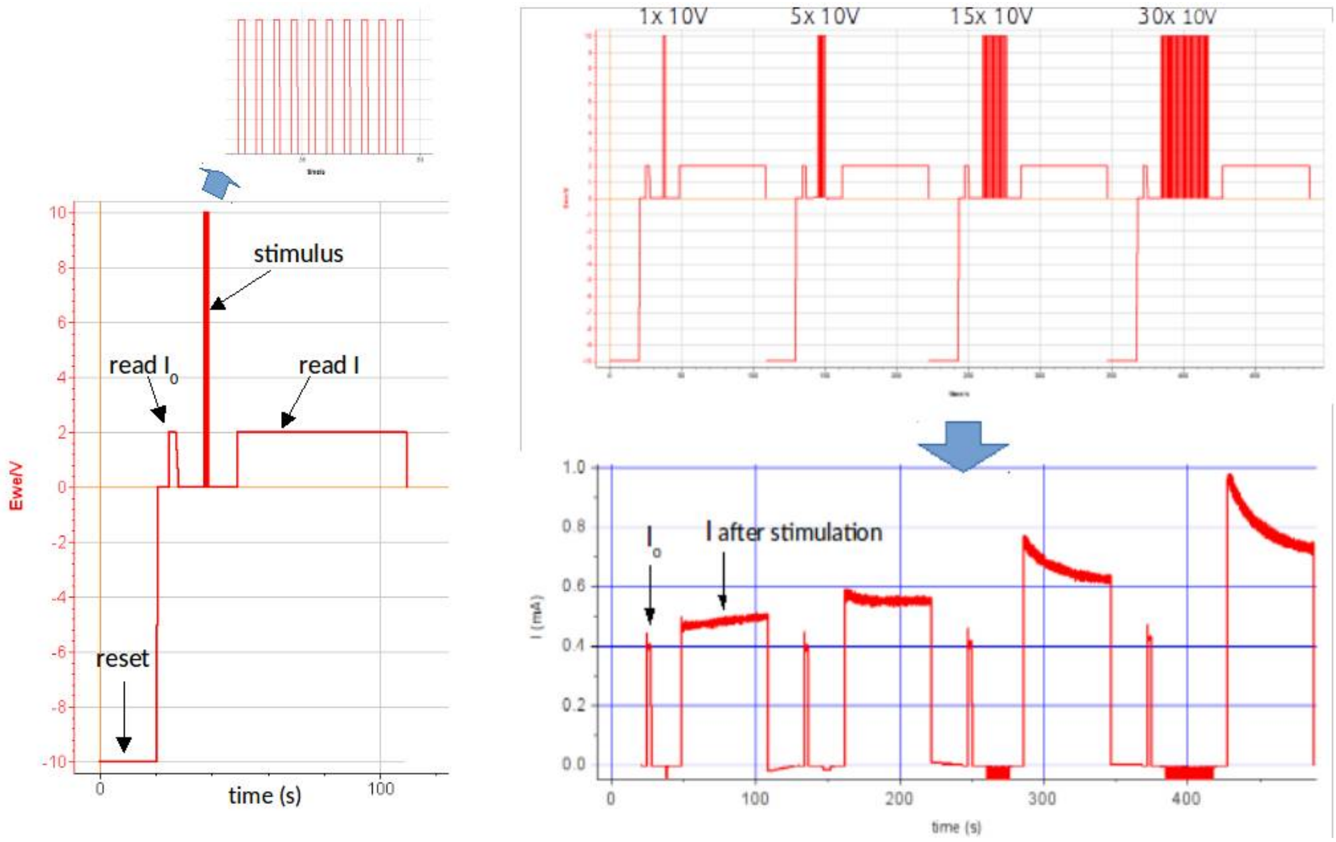

Supplement: Supplementary file 1 [file nanomaterials-09-00825-s001.pdf]
